# Primitive isolated hydatid cyst of the spleen: total splenectomy versus spleen saving surgical modalities

**DOI:** 10.1186/s12893-020-01036-8

**Published:** 2021-01-20

**Authors:** Atef Mejri, Khaoula Arfaoui, Mohamed Firas Ayadi, Badreddine Aloui, Jasser Yaakoubi

**Affiliations:** 1 General Surgery Department, Regional Hospital of Jendouba, Jendouba, Tunisia; 2 General Surgery Department, Mongi Slim Hospital, La Marsa, Tunisia

**Keywords:** Echinococcosis, Spleen, Surgery, Complication

## Abstract

**Background:**

This study aims to describe the clinical features of the isolated primitive splenic hydatid cyst, discuss and compare the different surgical approaches of this uncommon disease.

**Methods:**

This is a descriptive retrospective study carried out over a period of 7 years extending from January 2013 to December 2019 reporting eight cases of isolated primitive splenic hydatid cysts. Data were collected from the register of the general surgery department of the Jendouba regional hospital. Files concerning another associated hydatid localization were excluded. Four patients underwent total splenectomy and four of them underwent different spleen preserving surgical techniques including resection of the protruding dome, partial splenectomy and pericystectomy.

**Results:**

The diagnosis was incidentally made in 50% of cases. The main other revealing complaints are pain in the left upper quadrant of abdomen in 25% of cases and a painless renitent mass in the same quadrant in only 12,5%. None of patients who underwent total splenectomy had fever or sings of postoperative sepsis. Compared to those who had total splenectomy, patients who underwent spleen preserving surgery had a longer average hospital stay (9 vs 6,25 days) related to post-operative complications including abscess in the residual cavity after protruding dome resection in one patient and post-operative haemorrhage in one patient.

**Conclusions:**

The current case series argues in favor of total splenectomy, preferably by laparoscopic route whenever the technical platform allows it, associated with some specific peri-operative therapeutic measures. It seems to be the safest way that helps to avoid post-operative complications of spleen saving surgical modalities. These complications are usually difficult to manage in poor countries with limited technical resources. Total splenectomy guarantees at least a decreased hospital stay, reduced healthcare costs, and the absence of recurrence in highly endemic underdeveloped countries.

## Background

The splenic involvement in the hydatid disease is exceptional and the clinical presentation lacks specificity. Therefore, this cosmopolitan prasitosis should always be considered as a differential diagnosis of every splenic cyst. The aim of this study is to describe the clinical features, discuss and compare the different surgical modalities of this rare localization of the hydatid disease.

## Methods

This is a descriptive retrospective study carried out over a period of 7 years extending from January 2013 to December 2019 including hospitalized patients with confirmed abdominal hydatidosis. The retrospective analysis was done by collecting data from the register of the general surgery department of the Jendouba regional hospital and the clinical records of patients. Of the 391-hydatid disease files, only eight cases of isolated splenic localization were collected and all files concerning another associated hydatid localization were excluded. The various details of medical history, clinical examination, further examinations, treatment modalities and follow-up visits were reported. Once the diagnosis was established, all the patients underwent surgical treatment based on total splenectomy or another surgical method preserving the spleen adapted to each patient according to the clinical and radiological data. All surgical resection specimens were sent for pathological examination. Ethical approval for the study was obtained from the medical ethics committee of the Jendouba regional hospital.

## Results

**Fig. 1:**
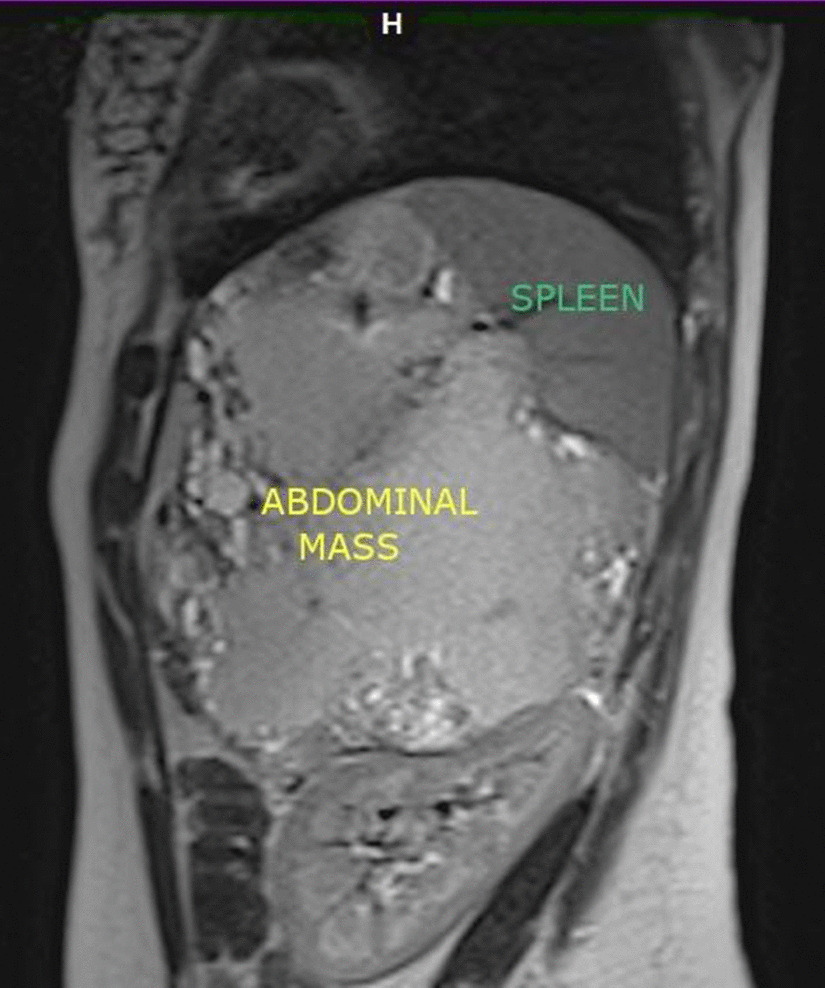
CT scan sagittal view showing a splenic hydatid cyst of 16 cm diameter

**Fig. 2:**
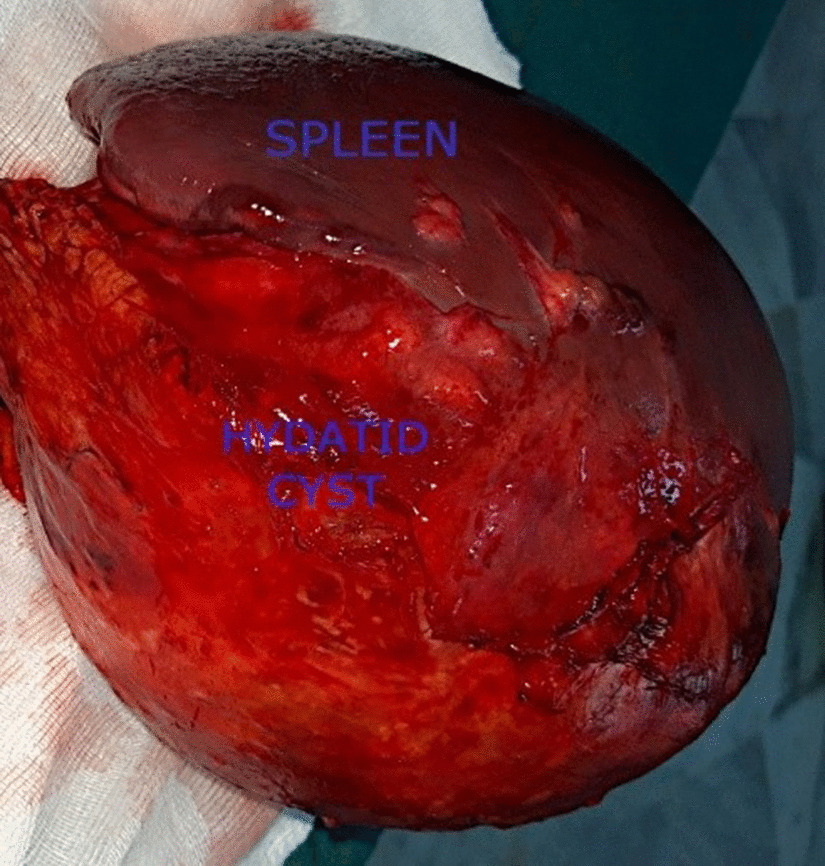
Intra operative view of a total splenectomy specimen

Among the eight patients involved in this study, there were three men (37.5%) and five women (62.5%). The average age was 42.62 years with extremes ranging from 17 to 62 years. Six patients lived in rural areas or had contact with cattle dogs. Pain in the left upper quadrant of the abdomen was the revealing symptom of the pathology in only two patients (25%). A patient presented with a painless renitent mass gradually increasing in size and located in the left upper quadrant of the abdomen. One patient always complained of dyspepsia with bothersome early postprandial fullness. For the four other patients, the discovery was fortuitous based on abdominal ultrasounds requested in the framework of assessment of biliary colic (2 patients), viral hepatitis B (1 patient) and renal colic (1 patient). Routine blood tests were normal in all patients. All patients had Hydatid serology using the enzyme-linked immunosorbent assays (ELISA) technique which was positive in only three cases (37.5%). Chest X ray failed to demonstrate any concomitant cysts in the lungs. All patients had abdominal ultrasound and abdominal CT. The cysts were solitary in 100% of cases. Their diameter varied from 2.9 to 16 cm with an average of 7,91 cm. According to Gharbi's classification, two of them were classified as type I (25%), one cyst as type II (12, 5%), four cysts as type III (50%) and one cyst as type IV (12, 5%). The location of the cyst was superior polar in one case (12, 5%), inferior polar in five cases (62, 5%), centro-parenchymal in two cases (25%). An anti-pneumococcal and anti-meningococcal vaccine was given to all patients approximately 15 days before surgery. All patients were operated using a left subcostal approach. Four patients had a total splenectomy for two giant cysts almost destroying all the spleen parenchyma and two cysts located near the hilum (Figs. 1 , 2). Two patients of the remaining group had a resection of the protruding dome for easily accessible cysts located on the surface of the spleen. One patient had partial splenectomy for an inferior polar cyst measuring 4 cm in diameter and the last patient had pericystectomy for a 2.9 cm diameter cyst (Table 1). Meticulous protection of the operating field using surgical swabs soaked with a scolicidal solution (20% hypertonic saline solution) was performed in all patients. One of the patients who had total splenectomy presented on postoperative day 5 with extreme reactive thrombocytosis with a platelet count exceeding 1000 K/µL requiring him to be put on Acetyl salicylic Acid with Enoxaparin therapy. The iterative check-up blood tests showed a decreased platelet count with a return to normal on post-operative day 12. The remaining three patients who had total splenectomy had a slight transient thrombocytosis with a platelet count below 650 K/µL. Oral antibiotic prophylaxis based on phenoxymethylpenicillin (1 g/day) was administered to all patients undergoing total splenectomy. None of them had fever or sings of postoperative sepsis. One of the patients treated with protruding dome resection leaving behind a non-declivitous residual cavity had fever on post-operative day five associated with vomiting and a biological inflammatory syndrome. An abdominal CT scan performed the same day revealed an abscess occupying this cavity. The decision was to combine CT-guided percutaneous drainage and intravenous antibiotic therapy with a good outcome. For the other patient, the residual cavity left was declivitous and the postoperative course was uneventful. The patient who underwent partial splenectomy had previous systemic hypertension. On postoperative day 1, his blood pressure remained in the normal range but he was mildly tachycardic. The hemoglobin did not decrease and 75 ml sanguineous fluid was collected during the preceding 24 h via the Redon suction drain. A CT scan performed within 12 h after the onset of the symptoms showed a hemoperitoneum in the perisplenic space and the Douglas pouch without active extravasation of arterial contrast enhanced agent. A revision surgery was conducted on postoperative day 2 to improve hemostasis and perform peritoneal lavage with drainage. The postoperative outcome was good. Scrupulous wound care helped prevent wound infections in all patients. The hospital stay ranged from 5 to 13 days with an average of 7.6 days. Pathological examination confirmed the hydatid disease in all cases. All patients received Albendazole (15 mg/kg/day) for 3 months after surgery. Mean follow-up period was 38 months. No case of recurrence was observed till now.

**Table 1:** The main clinical features corresponding to each hydatid cyst

Patient	Sex	Age	Discovery	Cyst location	Gharbi classification	Maximal diameter (cm)	Surgical technique	Post-operative complication requiring surgical/percutaneous treatment
1	Male	17	Dyspepsia	Inferior polar	Type III	7.2	Protruding dome resection	None
2	Male	54	Incidental (Renal colic)	Inferior polar	Type II	4	Partial splenectomy	Hemorrhage
3	Male	62	Abdominal pain Left upper quadrant of abdomen	Superior polar	Type I	5.7	Protruding dome resection	Abscess in the residual cavity
4	Female	46	Incidental (biliary colic)	Centro-parenchymal	Type III	7.5	Total splenectomy	none
5	Female	39	Palpable Mass Left upper quadrant of abdomen	Inferior polar	Type III	14	Total splenectomy	None
6	Female	43	Abdominal pain Left upper quadrant of abdomen	Inferior polar	Type IV	16	Total splenectomy	None
7	Female	29	Incidental (Hepatitis B)	Inferior polar	Type I	2.9	Pericystectomy	None
8	Female	51	Incidental (biliary colic)	Centro-parenchymal	Type III	6	Total splenectomy	None

## Discussion

Tunisia is a North African country with wide sheep grazing areas, making it endemic for hydatid disease. Spleen involvement in hydatid disease is relatively rare even in endemic zones [1]. The isolated and primitive spleen localization of the hydatid disease is much more exceptional, representing only 2 to 3,5% of cases [2–4]. This corroborates with data in the current study showing that isolated primary splenic localization represents only 2, 04% of abdominal hydatid disease. The mechanisms responsible of splenic involvement may clarify the rarity of this entity. Only the minority of Echinococcus Granulosus eggs that manage to escape both hepatic and pulmonary barriers may reach the spleen via systemic route [5]. Portal, lymphatic and colonic trans-parietal passage are other infrequent contamination routes that explain the disease [6–8].

The very slow growth rate (0,3 cm to 1 cm per year) explains the long period of clinical latency [9], which can reach up to 20 years [3, 10]. In our study, the average age of diagnosis was over 40. It also explains the fact that in many studies including the current one, the fortuitous discovery is a considerable revealing circumstance [6, 11]. The main clinical symptoms are dominated by left upper quadrant abdominal pain [6, 11] (25% in our series) and a palpable mass in the left upper quadrant which is the other most common.

Complaint [10]. The diagnosis may also be suspected based on signs reflecting complications such as infection of the cyst, pressure effects, fistula formation or rupture to neighboring organs and structures [5, 11, 12]. That may include fever, dyspepsia, dyspnea, pleural effusion, constipation, generalized abdominal pain or signs of anaphylactic choc which is undoubtedly a life-threatening condition related to cyst spontaneous or traumatic rupture [5, 7]. Garg et al. reported a unique case of isolated hydatid cyst in the inferior pole of the spleen ruptured into the splenic flexure of colon causing unusual signs of chronic diarrhea and emaciation [7].

Physical examination reveals in most cases, nothing except a painless splenomegaly [2, 6].

The routine blood tests are often normal. Hydatid serology is contributive in extrahepatic localizations in only 65% of cases [9, 10]. In our series it was associated with a false negative rate of 62,5%. Therefore, it represents an unreliable diagnostic clue. Ultrasound is the best first-line examination for the diagnosis of hydatid cyst of the spleen thanks to its sensitivity of 90–95% [8, 10, 13]. The first ultrasound classification is the one developed by Gharbi et al. since 1981 allowing a standardized classification following the natural history of the parasite [8].

Abdominal CT, with a higher sensitivity (95% to 100%), has revolutionized the management of this disease. It can directly identify the cyst, specify its size, location and detailed anatomic contacts with surrounding vessels and organs, and it can look for other concomitant intra-abdominal localizations and evaluate possible complications [10, 12, 13]. The diagnosis is strongly guided by some suggestive signs such as parietal calcifications, floating membrane, daughter cysts and the presence of other concomitant extra-splenic cystic images [11, 14]. The CT is also a more powerful imaging tool used to detect recurrences [10, 13]. The diagnostic accuracy of the combination of Ultrasonography and CT is 100% in our series. This high diagnostic value expresses further support for the idea of other series [15] that US-CT combination is the most useful diagnostic procedure.

The treatment of hydatid spleen cysts remains primarily surgical [5, 16]. The therapeutic modalities vary between total splenectomy allowing a radical treatment of the parasite and spleen-saving methods including deroofing with omentoplasty, pericystectomy, partial splenectomy, and internal cysto-jejunal anastomosis [3, 12].

Total splenectomy certainly allows avoiding recurrences and complications related to the residual cavity [5, 16]. However, in addition to its technical difficulty due to adhesions to neighboring organs induced by chronic pericystic inflammation [11, 12], the risk of gastric or pancreatic injuries and sometimes the difficulty of splenic pedicle control [2], it exposes to overwhelming post splenectomy infections (OPSI) and thromboembolic complications [17].

The related mortality rate ranges to 1.9% in adults and 4% in children [5, 10]. For these reasons, authors recommend keeping this method only for multiple cysts [9, those occurring on a pathological spleen, those not offering a clear protruding dome [9, 11], centroparenchymal cysts [9, 11], the cysts located near the splenic hilum [12] and those with adhesions to surrounding organs [18]. Total splenectomy is also the treatment of choice for giant splenic cysts destroying more than 75% of the spleen parenchyma [9, 11] because in this case the splenic parenchyma is very reduced and almost completely destroyed by the pericystic fibrosis [10]. In the current series, the giant volume of the cyst destroying almost all of the splenic parenchyma made it necessary to choose this method in two cases. Whereas in the two other cases, the cysts were very close to the spleen hilum.

As for the spleen- saving approaches, they are dominated by deroofing and omentoplasty, which is a simple classical conservative technique described first by Lagrot. It is suitable for polar cysts with an accessible protruding dome or in the presence of adhesions between the spleen and the surrounding organs making total splenectomy risky [9]. It is associated with a low risk of hemorrhage compared to partial splenectomy, however, it exposes to the risk of postoperative suppuration due to the persistence of a residual cavity [8]. This risk is considerable (25% in our study) and it cannot be overlooked. Whereas, total splenectomy allows avoiding this major disadvantage. Similarly to some studies that showed no significant difference in term of recurrence between this surgical procedure and total splenectomy [10, 19], our series showed no recurrence case among all patients. Partial splenectomy and pericystectomy are other alternatives that make it possible to alleviate the risk of septic post-operative complications by preserving the immune function of the spleen. Partial splenectomy is suitable for polar cysts and requires leaving behind at least 25% of the splenic tissue [11]. However, it exposes to a significant hemorrhagic risk [10] especially in adults [20] as shown in the current study. The hospital stay is clearly extended with not went to plan conservative surgical methods (Table 2).

**Table 2:** average hospital stay for total splenectomy and spleen preserving modalities

Surgical technique	The average hospital stay (day)
Total splenectomy	6.25
Conservative methods	9

Punction aspiration injection and reaspiration (PAIR) is an emerging curative option that has been increasingly advocated due to its minimally invasive nature. It is known to be a safe procedure with much shorter hospital stay and lower morbidity than total splenectomy [18]. It also offers a good alternative to surgery for patients with high anesthesia risk [9, 10]. However, it can be proposed only for simple [10] type I or II [3, 9] and small cysts (< 5 cm) [3, 9, 11].

Whichever technique is chosen, the laparoscopic approach remains an increasingly advocated method due to its minimally invasive nature and its good results [8, 21]. While other authors have reservations about such an approach because of the risk of accidental cystic rupture with an anaphylactic shock, which can be a source of spillage of cystic fluid and postoperative secondary peritoneal echinococcosis [8, 9]. Sharma recommend laparoscopic approach only for small splenic hydatid cysts [22]. According to others, it is also not recommended to choose laparoscopic surgery in case of multiple [10, 21] or infected cysts [10]. Moreover, this surgical modality is sometimes limited by the high cost of training and equipment and the high surgical skills needed.

Albendazole-based perioperative therapy is recommended for at least one month before and after the surgical procedure in order to reduce the volume of the cyst to facilitate its resection and to reduce the risk of postoperative secondary hydatid cysts by sterilizing the disseminated protoscoleces [15, 19].

## Conclusion

Despite the fact that many authors from all over the world have currently become a little reluctant to perform total splenectomy because of the risks that are incurred by this procedure and dominated by some serious postoperative infections, this method remains an option of choice especially in underdeveloped countries like ours.

Indeed, the technical platforms available there do not offer the possibility to easily perform more sophisticated surgical methods such as laparoscopic or robotic partial splenectomy, pericystectomy or protruding dome resection in the daily surgical routine.

In addition, the management of immediate postoperative complications of conservative methods such as hemorrhage or abscess formation in the residual cavity is difficult because of the unavailability of interventional radiology thus, requiring another surgical intervention as demonstrated in this series. All these reasons incite the surgeon to approach the subject from a different angle and they argue in favor of total splenectomy especially in adults. Total splenectomy associated with some crucial and mandatory perioperative therapeutic measures, including preoperative vaccination, subcutaneous prolonged postoperative anticoagulant therapy, antibiotherapy, complete blood count twice a week, thoughtful surveillance and adjusted care according to each patient’s associated illnesses, seems to be the most convenient choice for highly endemic and poor countries with often limited technical resources. It guarantees at least a decreased hospital stay, reduced healthcare costs, and the absence of recurrence. Laparoscopic route for total splenectomy should always be advocated as it has the advantages of minimizing postoperative pain, large wound infections, postoperative complications related to bed rest and hospital stay period.

## Data Availability

The datasets used and/or analysed during the current study are available from the corresponding author on reasonable request.

## Abbreviations

ELISA: Enzyme-linked immunosorbent assays
CT: Computed tomography
OPSI: Overwhelming post splenectomy infections
PAIR: Punction aspiration injection and reaspiration
